# Supplementary material for: Speed of environmental change frames relative ecological risk in climate change and climate intervention scenarios
Source: Nat Commun. 2024 Apr 18;15:3332. doi: 10.1038/s41467-024-47656-z (PMC11026408; doi:10.1038/s41467-024-47656-z)
Supplement: Supplementary file 1 — Supplementary Information [file 41467_2024_47656_MOESM1_ESM.pdf]

Supplementary Information for: Speed of environmental change frames relative ecological risk in climate change and climate intervention scenarios

Daniel M. Hueholt[1*], Elizabeth A. Barnes[1†], James W. Hurrell[1†], Ariel L. Morrison[1]

[1*]Department of Atmospheric Science, Colorado State University, 200 West Lake Street 1371 Campus Delivery, Fort Collins, 80523, Colorado, United States of America.

*Corresponding author(s). E-mail(s): daniel.hueholt@colostate.edu; Contributing authors: eabarnes@colostate.edu; jhurrell@rams.colostate.edu; ariel.morrison@colostate.edu;
†These authors contributed equally to this work.

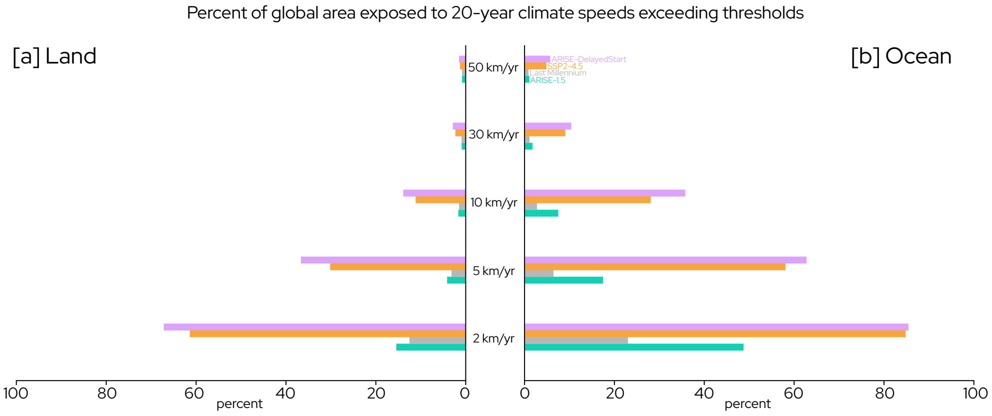

Supplementary Figure 1 Title: Percent of global land and ocean area exposed to threshold climate speeds
Percent of global land [a] and ocean [b] area exposed to 20-year ensemble mean climate speeds beyond selected threshold values in Shared Socioeconomic Pathway 2-4.5 (SSP2-4.5), Last Millennium, and Assessing Responses and Impacts of Solar climate intervention on the Earth system (ARISE) 1.5 and DelayedStart simulations. Climate speeds are calculated over the ensemble mean of 2035-2054 (ARISE-1.5), 2045-2064 (ARISE-DelayedStart and SSP2-4.5), and the mean of ten 20-year periods (Last Millennium). Colors visually distinguish different datasets.

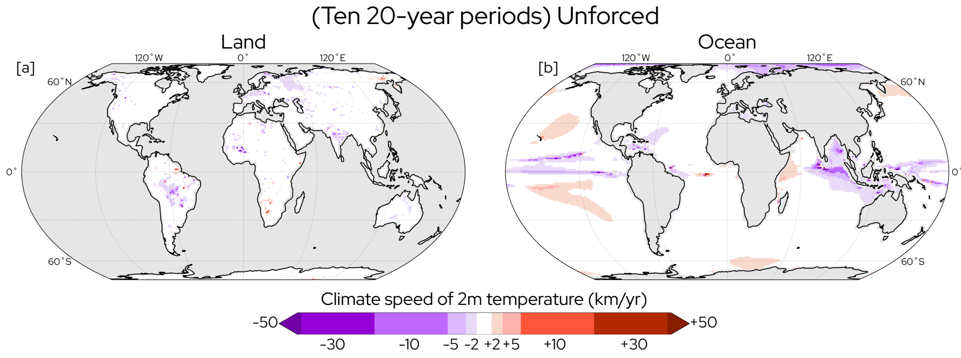

Supplementary Figure 2 Title: Climate speeds in Unforced simulation
Climate speeds in the mean of ten 20-year periods (to match ensemble size of other simulations, see Methods) in the Unforced simulation. The sign indicates whether the change in temperature associated with the climate speed is positive or negative. See Supplementary Fig. 8 for maps for each interval. Masked area shown in gray (ocean for [a], land for [b]).

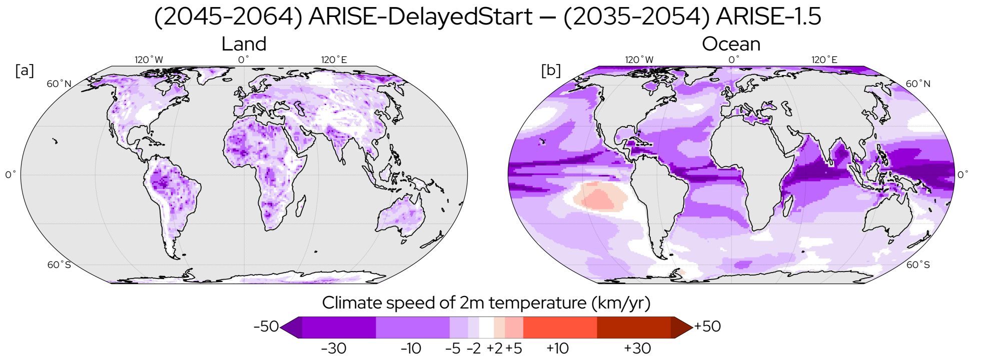

Supplementary Figure 3 Title: Difference of 20-year climate speeds following stratospheric aerosol injection deployments
Climate speeds during the 20-year period immediately following deployment of stratospheric aerosol injection (SAI) on land [a] and ocean [b] in the ensemble mean of Assessing Responses and Impacts of Solar climate intervention on the Earth system (ARISE) DelayedStart minus the ARISE-1.5 simulation. The sign indicates whether the change in temperature associated with the climate speed is positive or negative. Masked area shown in gray (ocean for [a], land for [b]).

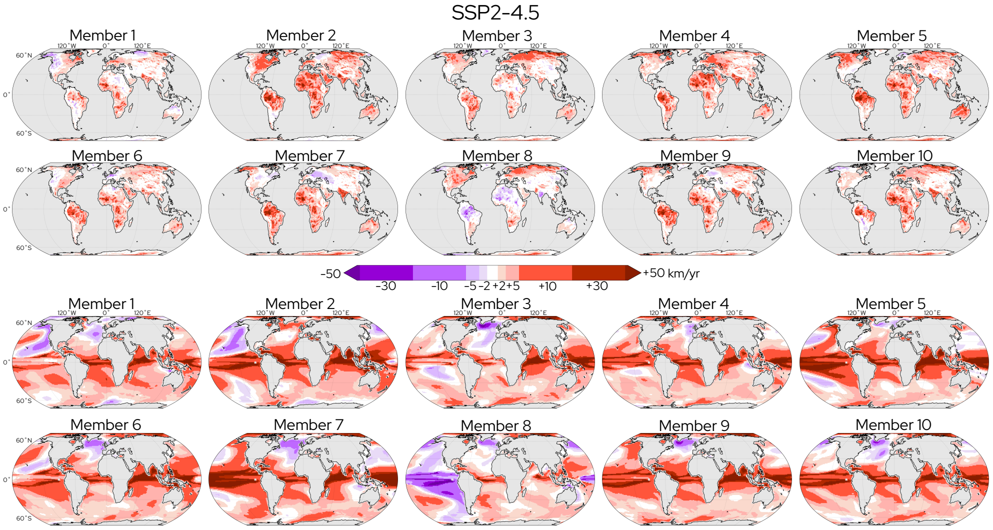

Supplementary Figure 4 Title: 20-year climate speeds by ensemble member for Shared Socioeconomic Pathway 2-4.5 (SSP2-4.5)
20-year climate speeds (2045-2064) for land (top half) and ocean (bottom half) in each of the ten ensemble members of Shared Socioeconomic Pathway 2-4.5 (SSP2-4.5). Masked area shown in gray (ocean for top half, land for bottom half).

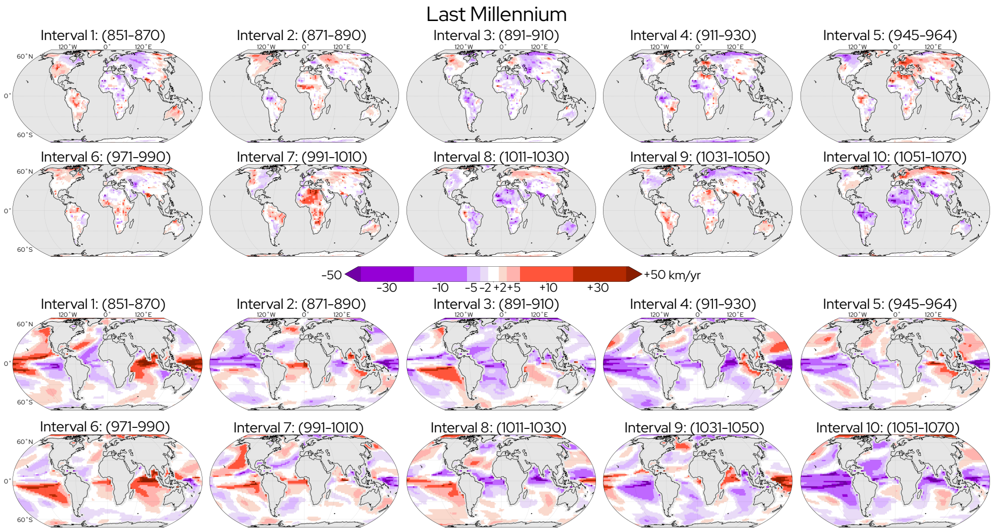

Supplementary Figure 5 Title: 20-year climate speeds by interval for Last Millennium
20-year climate speeds for land (top half) and ocean (bottom half) in each of the ten intervals treated as different ensemble members in the Last Millennium simulation for Figures 1, 3, and Supplementary Fig. 1. Masked area shown in gray (ocean for top half, land for bottom half).

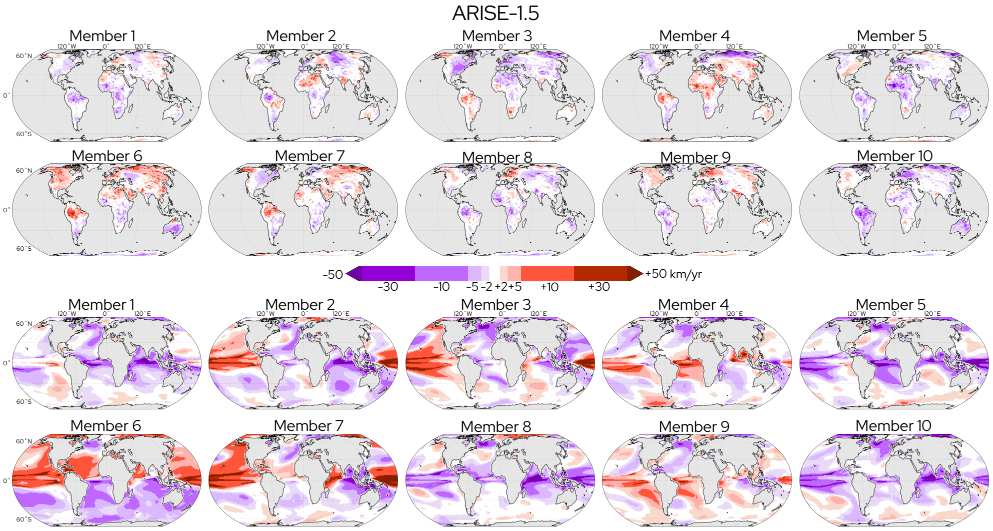

Supplementary Figure 6 Title: 20-year climate speeds by ensemble member for Assessing Responses and Impacts of Solar climate intervention on the Earth system-1.5 (ARISE-1.5)
20-year climate speeds (2035-2054) for land (top half) and ocean (bottom half) in each of the ten ensemble members of the Assessing Responses and Impacts of Solar climate intervention on the Earth system-1.5 (ARISE-1.5) simulation. Masked area shown in gray (ocean for top half, land for bottom half).

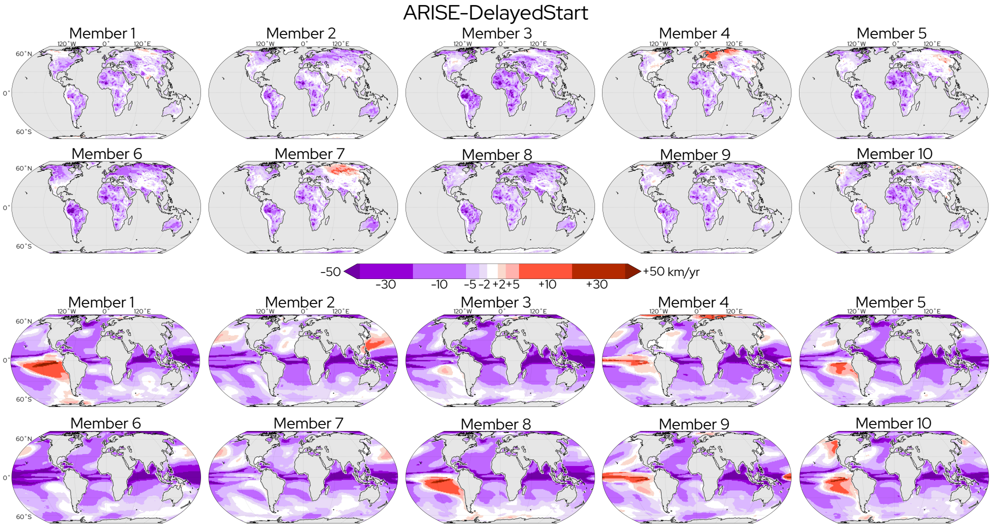

Supplementary Figure 7 Title: 20-year climate speeds by ensemble member for Assessing Responses and Impacts of Solar climate intervention on the Earth system-DelayedStart (ARISE-DelayedStart)
20-year climate speeds (2045-2064) for land (top half) and ocean (bottom half) in each of the ten ensemble members of Assessing Responses and Impacts of Solar climate intervention on the Earth system-DelayedStart (ARISE-DelayedStart) simulation. Masked area shown in gray (ocean for top half, land for bottom half).

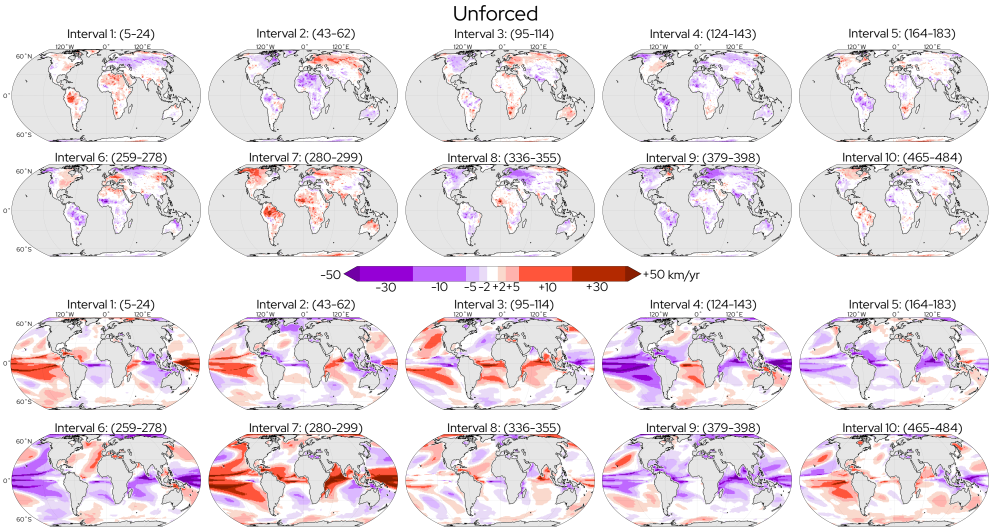

Supplementary Figure 8 Title: 20-year climate speeds by interval for Unforced
20-year climate speeds for land (top half) and ocean (bottom half) in each of the ten intervals treated as different ensemble members in the Unforced simulation for Supplementary Fig. 2. Masked area shown in gray (ocean for top half, land for bottom half).

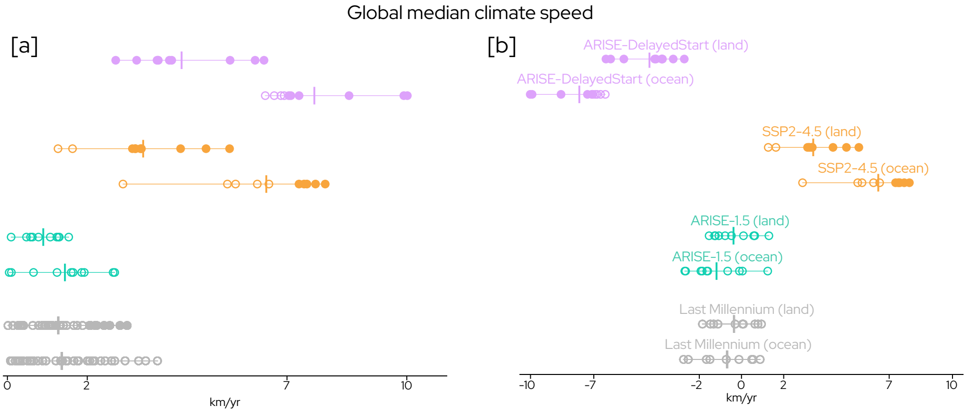

Supplementary Figure 9 Title: Variability of global median climate speeds
Global median climate speeds of 2m temperature over land and ocean in Shared Socioeconomic Pathway 2-4.5 (SSP2-4.5), Last Millennium, and Assessing Responses and Impacts of Solar climate intervention on the Earth system (ARISE) 1.5 and DelayedStart simulations. Open circles denote climate speeds with magnitudes within the mean dispersal speed of terrestrial or ocean species, closed circles signify climate speeds with magnitude exceeding mean dispersal speeds, and vertical bars show the ensemble mean. In [a], climate speeds are calculated over 2035-2054 (ARISE-1.5), 2045-2064 (ARISE-DelayedStart and SSP2-4.5), and every non-overlapping 20-year period avoiding large volcanic eruptions (Last Millennium). In [b], climate speeds are calculated over 2035-2054 (ARISE-1.5), 2045-2064 (ARISE-DelayedStart and SSP2-4.5), and ten 20-year periods avoiding large volcanic eruptions (Last Millennium) and illustrate both the sign and magnitude of the responses. Colors visually distinguish different datasets.

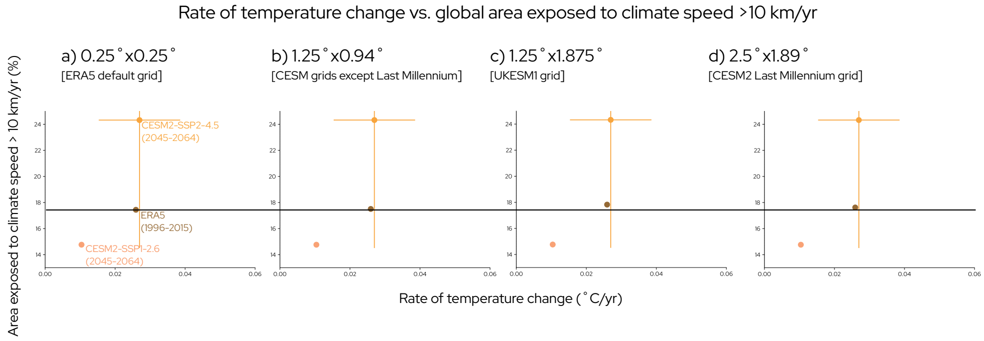

Supplementary Figure 10 Title: The influence of grid resolution on climate speeds
20-year rate of temperature change per year vs. percent of area exposed to a climate speed with magnitude greater than 10 km/yr, demonstrating the influence of grid resolution on the calculated climate speed in European Reanalysis 5 (ERA5). Horizontal black bar provides reference line of constant area exposed to provide greater clarity of the subtle difference between the three figures. Community Earth System Model 2-Shared Socioeconomic Pathway 1-2.6 (CESM2-SSP1-2.6) and CESM2-SSP2-4.5 shown for visual context. See Table 1 and Methods for detailed descriptions of each dataset. Colors visually distinguish different datasets.

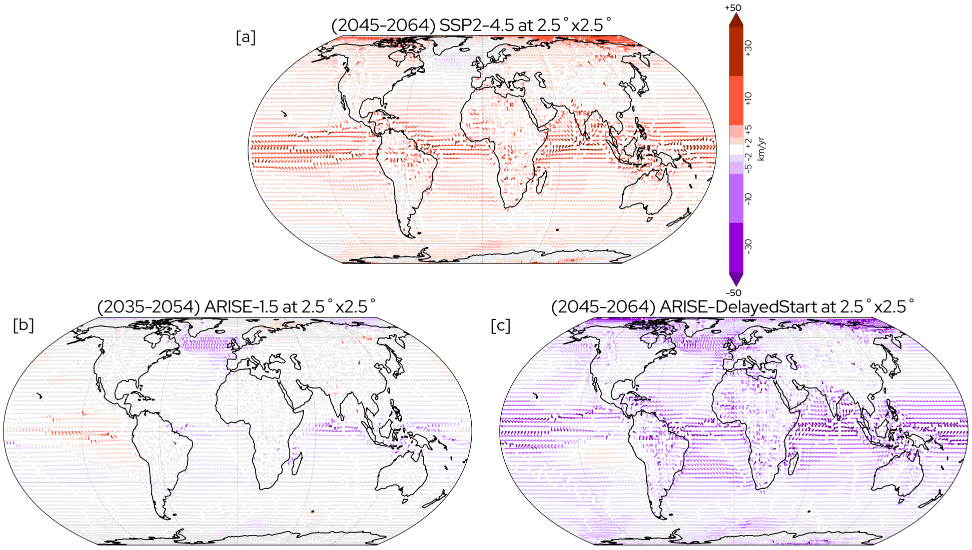

Supplementary Figure 11 Title: 20-year climate velocity vectors of 2-meter temperature
20-year climate velocities in the ensemble mean for Shared Socioeconomic Pathway 2-4.5 (SSP2-4.5) [a], Assessing Responses and Impacts of Solar climate intervention on the Earth system 1.5 (ARISE-1.5) [b], and ARISE-DelayedStart [c] simulations. The sign indicates whether the change in temperature associated with the climate velocity is positive or negative. Data regridded to 2.5°x2.5° for visual clarity.
